# Def Functions as a Cell Autonomous Factor in Organogenesis of Digestive Organs in Zebrafish

**DOI:** 10.1371/journal.pone.0058858

**Published:** 2013-04-12

**Authors:** Ting Tao, Hui Shi, Delai Huang, Jinrong Peng

**Affiliations:** Key Laboratory for Molecular Animal Nutrition, Ministry of Education, College of Animal Sciences, Zhejiang University, Hangzhou, Zhejiang Province, China; Institute of Cellular and Organismic Biology, Taiwan

## Abstract

Digestive organs originate from the endoderm. Morphogenesis of the digestive system is precisely controlled by multiple factors that dictate the cell fate and behavior so that the specific digestive organs are timely formed in the right place and develop into right size and structure. We showed previously that *digestive organ expansion factor* (*def*) is a gene whose expression is enriched in the liver, pancreas and intestine. Loss-of-function of *def* in the *def^hi429^* mutant confers hypoplastic digestive organs partly due to alteration of expression of genes related to the p53 pathway. However, the molecular mechanism for the involvement of Def in the organogenesis of digestive organs is still largely unknown. For example, it is not known whether Def regulates specific pathways in a specific organ. To address this question, we generated four independent *Tg(fabp10a:def)* transgenic fish lines which over-expressed Def specifically in the liver. We characterized *Tg-I*, one of the transgenic lines, in detail with genetic, molecular and histological approaches. We found that *Tg-I* restored the liver but not exocrine pancreas and intestine development in the *def^hi429^* mutant. However, *Tg-I* adult fish in the wild type (WT) background exhibits reduced liver-to-body ratio and all four transgenic lines conferred abnormal intrahepatic structure. Microarray data analysis showed that certain specific functional pathways were affected in the liver of *Tg-I*. These results demonstrate that Def functions in a cell autonomous manner during early liver development and aberrant Def protein expression might lead to disruption of the structural integrity of a normal adult liver.

## Introduction

Digestive system is formed by digestive tract and accessory organs such as liver and exocrine pancreas [1]. Zebrafish has been proven to be an excellent model system for the study of molecular control of organogenesis of digestive organs [2]. As in other vertebrate, zebrafish digestive system originates from the endoderm, one of the three germ layers defined during gastrulation [3]. Organogenesis of zebrafish digestive organs is controlled by spatially and temporally expressed endodermal factors and mesodermal signaling molecules [4]–[10]. We previously reported that Def was a pan-endodermal enriched factor that is essential for the growth of digestive organs in zebrafish [11]. Def is an evolutionally conserved molecule across eukaryotes from human to yeast [11]. Recent studies showed Def homologs in *Saccharomyces cerevisiae* (Utp25p) [12], [13] and *Arabidopsis thaliana* (NOF1) [14] are nucleolus-localized proteins and regulate pre-rRNA processing as the component of small subunit (SSU) processome. The nucleolus is a subnuclear structure that exhibits dynamic morphological changes during cell cycle. The nucleolus serves as the site for rRNA biosynthesis, processing and maturation, and also as the site for assembly of ribosome large and small subunit [15]. Therefore, disruption of the nucleolus function is normally detrimental to a cell [15]. Recently, evidence has shown that some nucleolar factors are also essential for organogenesis during embryogenesis. For example, loss-of-function of *wrd36* confers small eyes and hypoplastic digestive organs [16] and *bap28* mutation leads to neurodegeneration [17] in zebrafish.

In this work, we sought to address the question how Def, as a nucleolar factor, regulates organogenesis of digestive organs in zebrafish. We focused on a specific question: can Def's function in the liver be uncoupled from that in the exocrine pancreas and intestine? Does Def regulate specific functional pathways in a specific organ? To address this question, we generated four independent Def transgenic lines in that *def* expression was under the control of liver-specific promoter *fabp10a* [18]. We crossed *Tg-I*, one of the transgenic lines with *def^hi429^* mutant and found that only the liver but not intestine and exocrine pancreas development in the mutant was rescued to normal by *Tg-I*. Through comparing the global gene expression profiles we found that Def over-expression in the adult liver of *Tg-I* altered the expression of genes in specific functional pathways. Histology analysis revealed that the adult liver in all four transgenic lines suffered from disorganized intrahepatic structure. Our result shed lights on understanding how Def regulates organogenesis of digestive organs in zebrafish.

## Methods

### Ethics statement

This study did not involve non-human primates. All experiments described in this study were performed in full accordance with the guidelines for animal experiments released by the National Institute of Animal Health. This study is approved by the Animal Ethic Committee at Zhejiang University (ETHICS CODE Permit NO. ZJU2011-1-11-009Y).

### Zebrafish lines and maintenance

Zebrafish (*Danio rerio*) were raised and maintained in the standard Zebrafish Unit (produced by Aisheng Zebrafish Facility Manufacturer Company, Beijing, China) at Zhejiang University under a constant 14 hour on/10 hour off light cycle at 28°C. Zebrafish wild type AB strain was used in this study. The *def^hi429^* mutant line [19] was provided by Professor Nancy Hopkins at Massachusetts Institute of Technology (Cambridge, USA). The two pairs of primers derived from *lacZ* and *def* were used to genotype the *def^hi429^* mutant [11]. *Tg(fabp10a:def)* transgenic fish lines were generated through injection of the *fabp10a:def* plasmid DNA into zebrafish embryos. Primer pair *def*_Ex4_Fw336 (5′-GGACACCGATGAAGAGGACGGGA-3′) and *def*_Ex6_Rv730 (5′-AGGCCTGTCCGATGGCTGGA-3′) that spans intron IV and V of the *def* gene was used to genotype the *Tg(fabp10a:def)* transgenic lines.

### DNA constructs and microinjection of plasmid DNA

*def* full length cDNA was amplified by primer pair *Nhe*I-*def* (5′-TTCGCTAGCATGGGCAAAAGAAGGCGAGGAAAA-3′) and *def*-*BamH*I (5′-CGCGGATCCTCATGTGCTTTTCTCCTCCCCCGT-3′) according to the sequence information reported previously [11]. For construction of the *fabp10a:def* fusion DNA construct, zebrafish *def* full length cDNA tailed with SV40 polyadenylation (pA) signal was cloned downstream of a 2.8-kb 5′-flanking sequence of zebrafish *fabp10a* gene in the pEGFP-C1 vector [18], in such way the *fabp10a* promoter will drive *def* expression specifically in hepatocytes in the transgenic fish. The *fabp10a-def* DNA plasmid DNA was linearized by *Not*I and *Sfi*I, purified and adjusted to 500 ng/μl in ddH_2_O containing phenol red dye. Approximately 200 pl of the DNA solution was injected into the zebrafish fertilized eggs at one-cell stage.

For generation of transgenic fish using the *miniTol2* transposon system, the *fabp10a:def* fusion DNA fragment was cloned into the pDB739 vector between the left and right recognition boarders for Tol2 transposase. Tol2 mRNA was obtained by in vitro transcription of *pT3TS/Tol2* plasmid linearized by *Smal*I. One nanolitre of the plasmid DNA (50 ng/μl) and *Tol2* mRNA (50 ng/μl) mix was injected into the fertilized eggs at one-cell stage.

### DNA extraction and southern blotting analysis

Zebrafish genomic DNA was extracted from embryos with Genomic DNA Cell & Tissue Kit (Aidlab) using the protocol recommended by the manufacturer. 30 μg genomic DNA was digested with *EcoR*I, separated by electrophoresis in 0.6% agarose gel. Probe was Digoxigenin (DIG)-labeled and southern blot hybridization was performed according to the manufacturer's instructions (Roche Diagnostics). *def* probe was amplified by primers *def* probe_Fw301 (5′-GCTGAAGTTGAAGGTGATAGTGAA-3′) and *def* probe_Rv575 (5′-TCTTCATCCTGTTCCTGTTGTGCT-3′) using the *def* plasmid as the template.

### Whole-mount RNA *in situ* hybridization (WISH)

WISH was performed as described [11]. *def* full-length, *fabp10a*, *fabp2*, *trypsin* and *insulin* RNA probes were labeled with DIG and their sequences information was described previously [11]. Photos were taken under a Nikon AZ100 microscope.

### RNA extraction, northern blotting analysis and qPCR

Total RNA from different samples was extracted using TRIzol (Invitrogen) according to manufacturer's instructions. Probes were DIG-labeled and northern blot hybridization was performed according to the manufacturer's instructions (Roche Diagnostics). The 5′-ETS, ITS1 and ITS2 probes were as described previously [17]. For real-time quantitative PCR (qPCR), total RNA was treated with DNase I prior to reverse transcription and purified with RNeasy mini kit (Qiagen). First strand cDNA was synthesized using M-MLV Reverse Transcriptase (Invitrogen). The qPCR was performed on CFX96^TM^ Real-Time System (Bio-Rad) using SsoFast EvaGreen Supermix (Bio-Rad) according to the manufacturer's instructions. Primer pairs used for qPCR were listed in Table S1.

### Microarray hybridization and data analysis

We monitored the gene expression in samples using GeneChip® Zebrafish Genome Array (Affymetrix). The Affymetrix GeneChip® Zebrafish Genome Array can be used to study gene expression of over 14,900 *Danio rerio* genes.

Total RNA was first treated with DNase I and then purified through Qiagen RNeasy mini kit (Qiagen). RNA was quantified on the Nanodrop ND-1000 and RNA integrity was checked with Agilent 2100 Bioanalyzer. Samples were then amplified and labeled using the 3′ IVT Express Kit (Affymetrix) and hybridized with the GeneChip® Zebrafish Genome Array for 16 h. After hybridization, the arrays were then washed and stained with the Affymetrix fluidics station 450, followed by scanned with the Affymetrix 3000 7G plus scanner. Dat and Cel files were obtained by the AGCC software, and the CHP files were generated with MAS5.0 method by Affymetrix Expression Console. Raw data were imported into the Agilent GeneSpring GX 11.0 software for data analysis.

### Protein analysis and antibodies preparation

Zebrafish embryos were deyolked in ice-cold PBS by passing through a 21 gauge needle and then lysed in Tris lysis buffer (PH8.0, EDTA free) containing 1×Complete Protease Inhibitor Cocktail (EDTA-free, Roche) and 25 μM MG132 (Sigma) by passing through a 26 gauge needle. Liver lobes were dissected from the adult fish after anaesthesia, and lysed directly in the same Tris lysis buffer. Protein samples were stored at −20°C after denaturation.

Proteins were separated on SDS-PAGE gel and then transferred to PVDF membrane (Millipore). Membranes were subjected to western blotting analysis with appropriate antibodies. Chemiluminescent signals were detected with ECL solutions according to manufacturer's instructions (Pierce). A zf-Def rabbit polyclonal antibody was used as described [11]. A mouse monoclonal (38F3) antibody against human Fibrillarin (ab4566) was purchased from Abcam.

### Cryo-sectioning and immunofluorescence staining

Cryo-sectioning was performed as described [11]. For immunofluorescence staining of cryo-sections, the slides were immersed in 0.01 M citrate (pH 6.0) and boiled in a microwave oven for three times, each for 5 min with the purpose to expose the antigens. After overnight cooling at 4°C in the retrieval buffer, the slides were briefly washed in PBST and incubated with proper primary antibody, followed by Alexa Fluor conjugated secondary antibody (Invitrogen). The slides were then mounted with a mount medium containing DAPI (Vector), and the images were taken under a Leica TCS SP5 confocal microscope.

### LBR measurement and H&E (Hematoxylin and Eosin) staining

LBR was measured according to the method described previously [20]. H&E staining was performed as described [21].

## Results

### Generation of *Tg (fabp10a:def)* transgenic zebrafish

**Figure 1:**
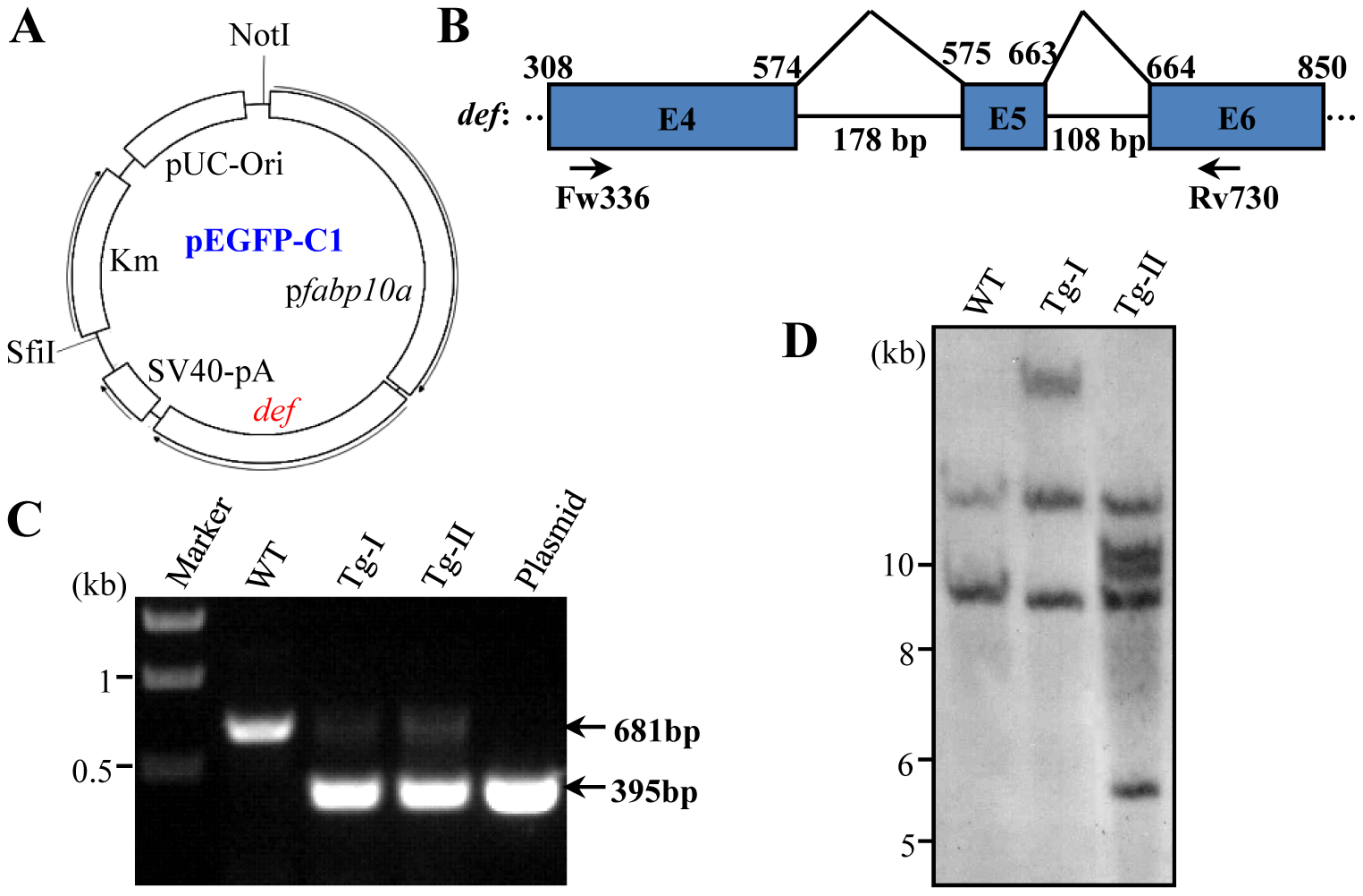
Generation of *Tg(fabp10a:def)* transgenic fish. (**A**) Diagram showing the *fabp10a:def* plasmid constructed in the pEGFP-C1. (**B**) Schematic drawing shows the genomic structure of the *def* gene from exon 4 to 6. Exon: blue box, intron: black line. Numerical number on the top indicates the cDNA nucleotide number beginning from the start codon ATG. Intron size was shown under the black line. Fw336 and Rv730 are the pair of primers used in PCR for screening of transgenic fish. (**C**) PCR products derived from the *Tg-I* and *Tg-II* transgenic fish. PCR products from the wildtype control fish (WT) and the *Tg(fabp10a:def)* plasmid were also shown. (**D**) Southern blotting analysis of the *def* transgene in *Tg-I* and *Tg-II*. DIG-labeled *def* cDNA was used as the probe.

The *fabp10a:def* plasmid DNA was linearized by *Not*I and *Sfi*I (Figure 1A) and was then injected into zebrafish fertilized eggs at one-cell stage. Three months later, these founders (F0 fish) were crossed with wild type (WT) fish. The progenies of each F0 fish were collected and pooled. Genomic DNA was extracted from the pooled embryos and used in PCR for screening for the germ line integration founders. Progenies (F1) from positive founders were allowed to grow to adulthood. The scales of each individual F1 fish were collected and lysed. The obtained DNA samples were used in PCR to identify the individuals harboring the transgene. WT fish is expected to produce a PCR product of a single band of 681 bp while a transgenic fish would yield a 395 bp band corresponding to the transgene that lacks the intron IV and V of the *def* gene (Figure 1B and C, lanes for WT and plasmid) in addition to the 681 bp band. Out of 37 founder fish, one line was identified to be a transgenic fish and was designated as *Tg-I* (Figure 1C, lane for Tg-I). We also obtained other three transgenic lines, including the line *Tg(fabp10a:def)-II* (*Tg-2*) (Figure 1C, lane for Tg-II), *Tg(fabp10a:def)-III* (*Tg-III*) and *Tg(fabp10a:def)-IV* (*Tg-IV*) (data not shown), through using the *miniTol2* transposon system.

We found that the progenies of *Tg-I* ^+/−^ × WT and of cross of male and female *Tg-I* ^+/−^ showed a 1∶1 and 1∶3 Mendelian segregation ratios (data not shown), respectively, suggesting that there is a single insertion in *Tg-I*. Southern blotting was performed to analyze the transgene in *Tg-I* and *Tg-II*. The result confirmed that *Tg-I* line harbored with a single insertion while *Tg-II* line with multiple insertions (Figure 1D).

### Def is over-expressed in the liver in *Tg-I*

**Figure 2:**
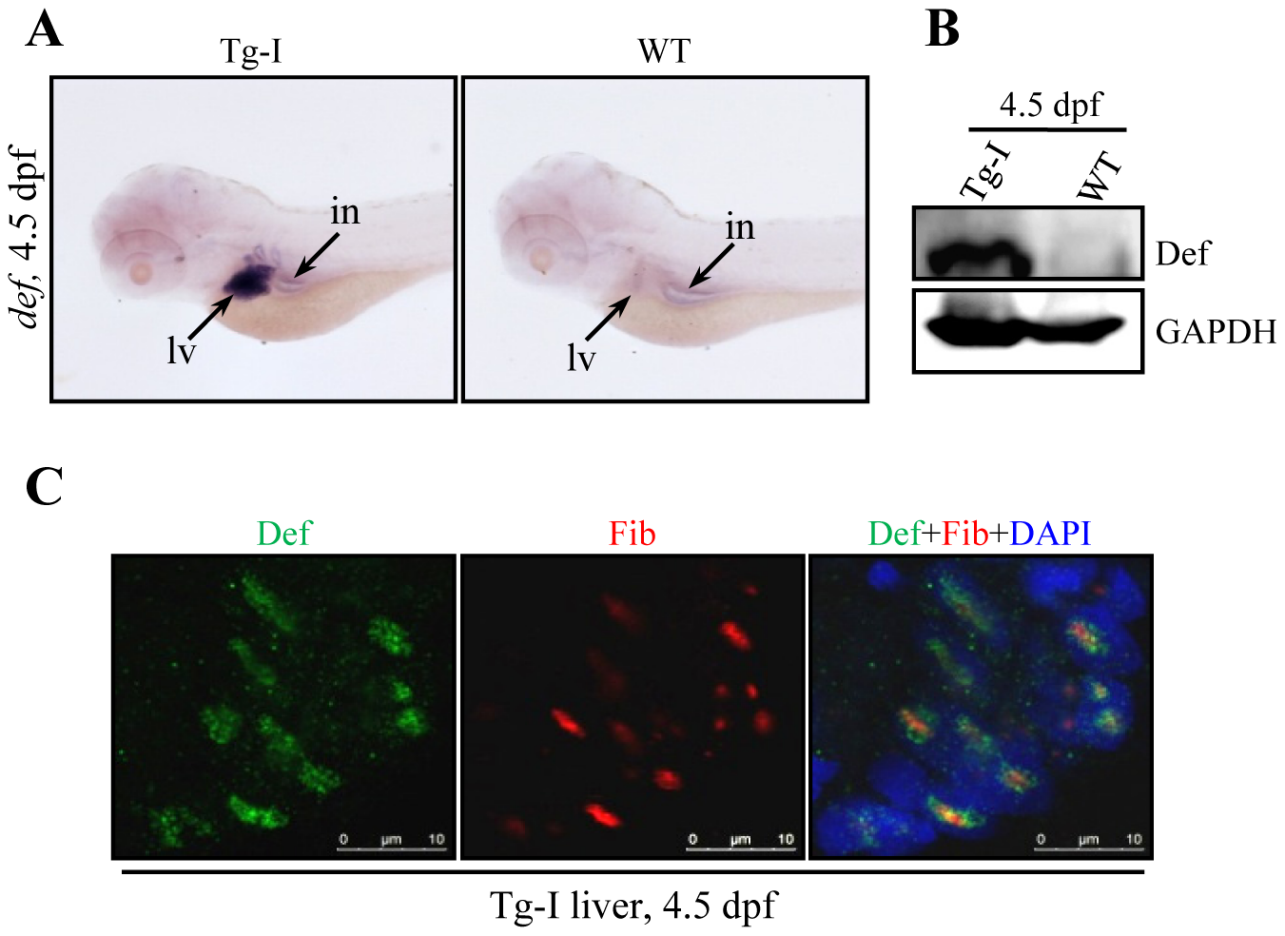
*Tg-I* expresses high level of *def* specifically in the liver. (**A**) WISH analysis of *def* expression in *Tg-I* and wildtype (WT) embryos at 4.5 dpf using the full length *def* cDNA as a probe. lv: liver, in: intestine. (**B**) Western blot of Def in *Tg-I* and wildtype (WT) embryos at 4.5 dpf. GAPDH: loading control. (**C**) Immunostaining of Def (green) and the nucleolar marker Fibrillarin (Fib) (red) in the liver of *Tg-I* fish at 4.5. DAPI was used to stain the nucleoli.

*fabp10a* is a liver specific gene whose expression is detectable in the hepatocytes from 2 dpf to adulthood in zebrafish [18], [22]. It is a relatively strong promoter which has been used to drive the reporter genes *EGFP* or *RFP* in previous studies [18], [23]. Since the *fabp10a* promoter is the most well characterized liver specific promoter in zebrafish [18] we used it to drive the expression of *def* in the hepatocytes. WISH was performed on the progenies derived from *Tg-I* with the DIG-labeled *def* full-length probe. We found that only those embryos harbored the transgene showed strong *def* expression specifically in the liver in a pattern similar to that did the *fabp10a* gene at 4.5 dpf [18], while the WT siblings exhibited weak endogenous *def* expression in the entire digestive organs (Figure 2A) as reported previously [11]. Western blot analysis with a Def polyclonal antibody showed that Def protein level in *Tg-I* was approximately 9-fold higher than that in the wild type control (Figure 2B). These evidences demonstrated that Def is over-expressed in *Tg-I*.

### Def protein is localized in the nucleolus

Def homologs in *Saccharomyces cerevisiae* (Utp25p) [12], [13] and *Arabidopsis thaliana* (NOF1) [14] have been shown to be localized in the nucleolus. We performed co-immunostaining of sections from 4.5 dpf old *Tg-I* fish with a Def antibody and an Fibrillarin (Fib, a nucleolar marker) antibody. The result showed that the over-expressed Def in the liver of *Tg-I* is co-localized with Fib within or at the periphery of the dense fibrillar component (DFC) regions of the nucleoli (Figure 2C).

### The *def* transgene in *Tg-I* rescues the liver but not intestine and exocrine pancreas development in *def^hi429^*

**Figure 3:**
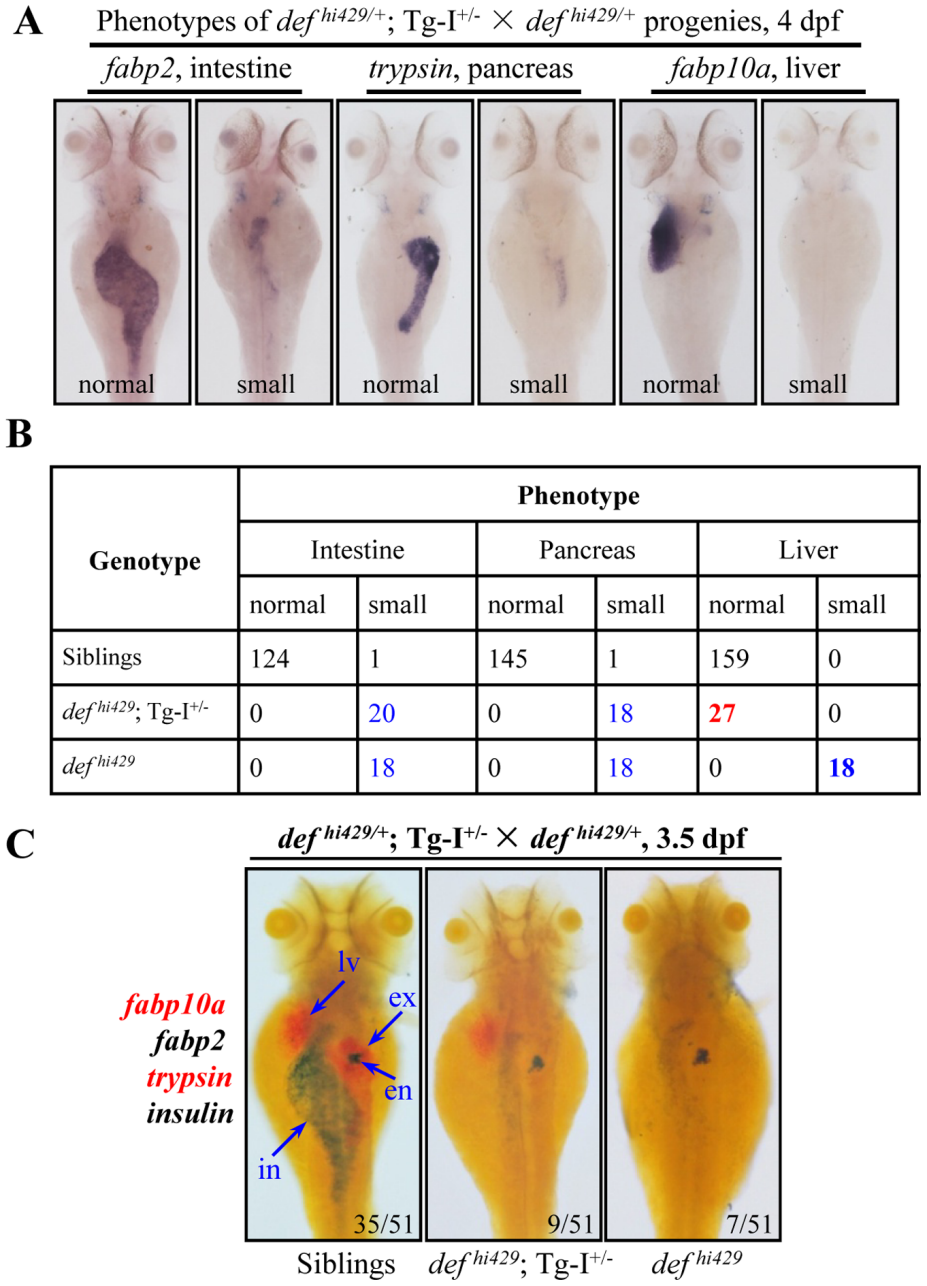
The *def* transgene in *Tg-I* rescues the liver but not intestine and exocrine pancreas development in *def^hi429^*. **(A, B**) WISH was performed to analyze the digestive organs in embryos at 4dpf produced by *Tg-I^+/−^ def^hi429/+^* × *def^hi429/+^* using the liver marker *fabp10a*, intestinal marker *fabp2* and exocrine pancreas marker *trypsin* (**A**). Embryos from (**A**) were then individually genotyped and matched to their phenotype. Number of embryos identified in each category was as shown in (**B**). (**C**) Progenies from *Tg-I^+/−^ def^hi429/+^* × *def^hi429/+^* at 3.5dpf were also examined simultaneously by Fast Red-labeled *fabp10a* and *trypsin* probes BCIP/NBT-labeled *fabp2* and *insulin* probes. Number of embryos in each category was obtained as shown after genotyping a total of 51 embryos from the cross. en: endocrine pancreas, ex: exocrine pancreas, in: intestine, lv: liver.

We showed in our previous report that *def* expression is enriched in the liver, exocrine pancreas and intestine but not in the endocrine pancreas. This expression pattern coincides with the hypoplastic phenotype of the liver, exocrine pancreas and intestine but with a normal endocrine pancreas exhibited by the *def^hi429^* mutant [11], suggesting that Def likely functions in a cell autonomous manner. In addition, our result showed that the development of the entire digestive system including liver, intestine and exocrine pancreas in the *def^hi429^* mutant could be restored to normal by *def* mRNA injection [11]. Apparently, the function of Def in a specific organ cannot be assessed by *def* mRNA injection into the *def^hi429^* mutant embryos since all cells will have equal chance to get the injected *def* mRNA. On the other hand, the *Tg-I* transgenic fish provided us with a unique chance because the *def* transgene is specifically expressed in the hepatocytes. We reckoned that if Def works in a cell autonomous manner the transgene will only be able to rescue the liver development but not the intestine and exocrine pancreas development in *def^hi429^*. To test this hypothesis, the *Tg-I* fish was first mated with *def^hi429^* heterozygous (*def^hi429/+^*) fish to get the *Tg-I^+/−^ def^hi429/+^* F1 fish. The progenies of *Tg-I^+/−^ def^hi429/+^* × *def^hi429/+^* were examined by WISH using the liver marker *fabp10a*, intestinal marker *fabp2*, and exocrine pancreatic marker *trypsin* (Figure 3A). All embryos were then genotyped individually and classified into three groups, namely siblings (including *Tg-I^+/−^*, *Tg-I^+/−^ def^hi429/+^*, *def^hi429/+^*, and wildtype), *Tg-I^+/−^ def^hi429^* double and *def^hi429^* single lines (Figure 3B). Data analysis revealed that the liver development in all *Tg-I^+/−^ def^hi429^* embryos was rescued by the *def* transgene (Figure 3B). In contrast, the *Tg-I def^hi429^* embryos still exhibited hypoplastic intestine and exocrine pancreas, same as that observed in *def^hi429^* (Figure 3B). We confirmed the above result by performing WISH to examine simultaneously the expression patterns of *fabp10a*, *fabp2*, *trypsin* and the endocrine marker *insulin* in the progenies of *Tg-I^+/−^ def^hi429/+^* × *def^hi429/+^* (Figure 3C). These genetic data demonstrate that 1) the *def* transgene in *Tg-I* is functional equivalent to the endogenous *def* gene, and 2) although Def expression is enriched in the entire digestive system it works in a cell autonomous manner.

### The *def* transgene continues to express at a high level in the adult liver

**Figure 4:**
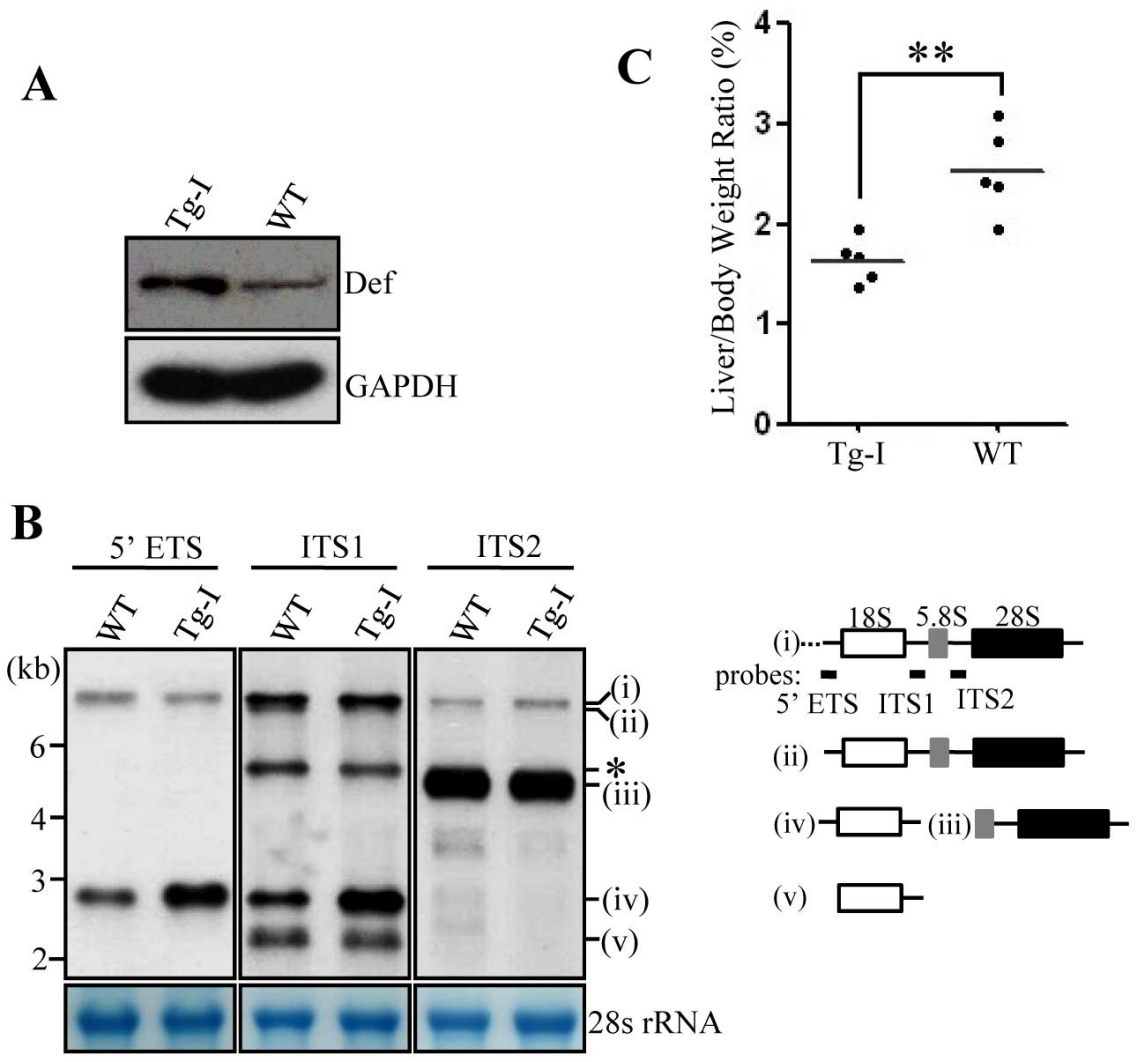
The liver of *Tg-I* confers an elevation of 18S rRNA precursor but a reduced LBR. (**A**) Western blot of Def in adult *Tg-I* and wildtype fish (3-month old). GAPDH: loading control. (**B**) Northern analysis of rRNA precursors using different probes as indicated. Probes used correspond to the positions highlighted in the drawing shown on the right. (**C**) Comparison of LBR in adult *Tg-I* and wildtype fish (3-month old). Data were collected from 5 WT and 5 *Tg-I* fish at 3-month old.

We followed expression of the *def* transgene in *Tg-I* to the adulthood. We first compared the transcriptional expression of *def* in the liver samples from three months old *Tg-I* and WT fish via qPCR. The result showed that *def* was expressed 268-folds higher in *Tg-I* than that in the WT fish. Western blot of Def in the liver samples showed that the level of Def protein was 3.3-fold higher in *Tg-I* than that in the WT fish (Figure 4A).

### The liver of *Tg-I* accumulates 18S rRNA intermediate precursor

Def is a nucleolar protein that has been found to regulate 18S rRNA processing in yeast and *Arabidopsis* [12]–[14]. Northern blot hybridization was performed to compare rRNA processing in the adult liver using probes detecting different rRNA precursors as indicated in Figure 4B (right panel). The result revealed that 18S rRNA precursor (Figure 4B, form iv as indicated, detected by 5′-ETS and ITS1 probes) but not 28S rRNA precursors (Figure 4B, form iii as indicated, detected by ITS2 probe) dramatically accumulated in the adult liver in *Tg-I*.

### The liver of *Tg-I* has a reduced LBR

We have shown that Def is essential for the expansion growth of digestive organs during the embryonic stage. We were intrigued to know whether Def over-expression in *Tg-I* will produce a larger liver. Zebrafish LBR is a sex but not age-dependent constant which has been used to evaluate liver growth by other researchers [20]. We measured the LBRs of 6-month-old WT and *Tg-I* male fish, respectively. To our surprise, the LBR in *Tg-I* is approximately 35.4% lower (p<0.01) than that in the WT fish (Figure 4C).

### Gene expression profiles were altered in the liver of *Tg-I*

**Figure 5:**
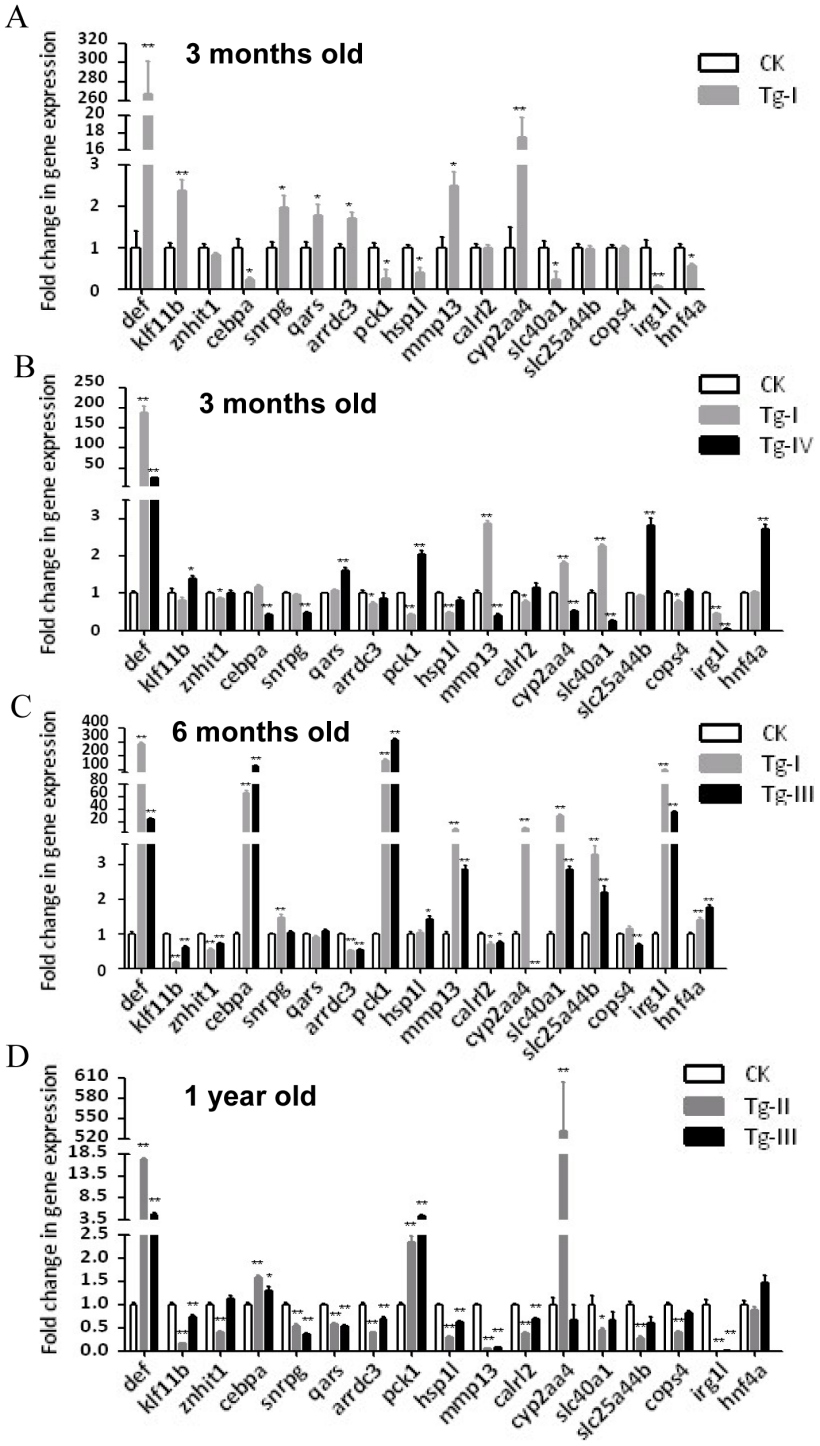
qPCR analysis of the expression of 16 differently expressed genes identified through microarray analysis in different transgenic lines. (A) Confirmation of microarray data by examining 16 genes selected from the list of up- or down-regulated genes based on our microarray analysis using total RNA extracted from 3 months old *Tg-I* liver. (B–D) Analysis of the expression of these 16 genes in different transgenic lines at different ages. Gene expression was analyzed by qPCR using gene specific primers and was expressed as fold change after normalization against *elf1a*. The value of CK was set as 1. CK: wild type adult liver control. *: *p*<0.05, **: *p*<0.01.

In view of LBR reduction in *Tg-I* we decided to perform microarray analysis with the purpose to find out whether Def over-expression will affect global gene expression profiles that might lead to LBR reduction. Total RNA from the adult liver of three independent batches of 3-month-old *Tg-I* and WT (AB strain) male fish was extracted using TRIzol (Invitrogen), each batch with the liver samples from three fish mixed together. Before subjecting to microarray hybridization, the RNA samples were confirmed to express dramatically high level of *def* by qPCR (data not shown). After data validation according to the procedure instructed by Affymetrix, the genes were filtered on flags that at least 3 out of 6 samples had values Present [P] or Marginal [M]. The logarithm base 2 of the signal ratio of *Tg-I* to WT <−1 or >1, as well as the unpaired *t*-test p value ≤0.05 were used as the cut-off value to identified candidate genes differently expressed in the liver in *Tg-I*. A total of 208 genes including *def* were identified (Table S2). After removing the same genes detected by redundant probe sets, a total of 202 genes were identified as differently expressed genes, of which 110 genes were up-regulated (Table S2) and 92 genes were down-regulated (Table S2). qPCR was performed to confirm the microarray data. Among 16 genes selected (selected from the list of genes with expression at least 2.5-fold higher or lower in *Tg-I* when compared to that in the WT control), expression of 12 genes (accounting for 75%) was confirmed to be up- or down-regulated in *Tg-I* (Figure 5A) by qPCR analysis.

Blast search revealed that 137 genes could be assigned a biochemical function while 65 belonged to unclassified, unknown or unnamed genes (Table 1 and Table S2). Gene ontology analysis classified the 137 genes into 11 categories. Among these 137 genes, 12 genes encode for transcription related proteins (including transcription factors, co-transactivators and transinhibitor), 12 for RNA binding and processing proteins (including RNA binding and splicing factors, RNA processing and ribosome biogenesis factors and tRNA synthetases), 44 for cell activity and structure related proteins (including cell cycle, proliferation, apoptosis, adhesion and migration proteins, energy metabolism, endocytosis and chromatin remodeling proteins, receptors, chaperones, cytokine, cytoskeleton related proteins and membrane proteins), 27 for various enzymes and proteinase inhibitor (including synthetase, synthase, hydratase, hydroxylase, dehydrogenases, decarboxylase, transferases, transaldolase, lyases, hydrolase, lysozyme, oxidase, reductase, protein kinases, protein phosphatase, pyrophosphatase, phospholipase, peptidase, mutase, metalloproteinase and proteinase inhibitor), 9 for ion binding proteins, 15 for carrier and transporter proteins, 3 for DNA binding and biosynthesis proteins, 6 for proteins involved in protein degradation, 9 for proteins involved in muscle function, neural function and immune response (Table S2).

**Table 1:** Classification of genes up- or down-regulated in the liver of *Tg-I*.

Protein category	Number of distinctive hits*
	(↑: up-regulated; ↓: down-regulated)
**Transcription related proteins**	
Transcription factors	2↑6↓
Co-transactivators	1↑2↓
Transinhibitor	1↑
**RNA binding and processing**	
RNA binding and splicing	3↑2↓
RNA processing and ribosome biogenesis	3↑1↓
tRNA synthetases	3↑
**Cell activity and structure**	30↑14↓
**Enzymes and proteinase inhibitor**	13↑14↓
**Ion binding**	7↑2↓
**Carriers and transporters**	5↑10↓
**DNA binding and biosynthesis**	1↑2↓
**Protein degradation**	3↑3↓
**Muscle function related**	1↑2↓
**Neural function related**	2↑1↓
**Immune response**	2↑1↓
**Unclassified**	4↑5↓
**Unknown and unnamed proteins**	29↑27↓

* Refer to Table S2 for details.

### Genes in specific functional pathways were down- or up-regulated in a coordinate way in the liver of *Tg-I*

**Figure 6:**
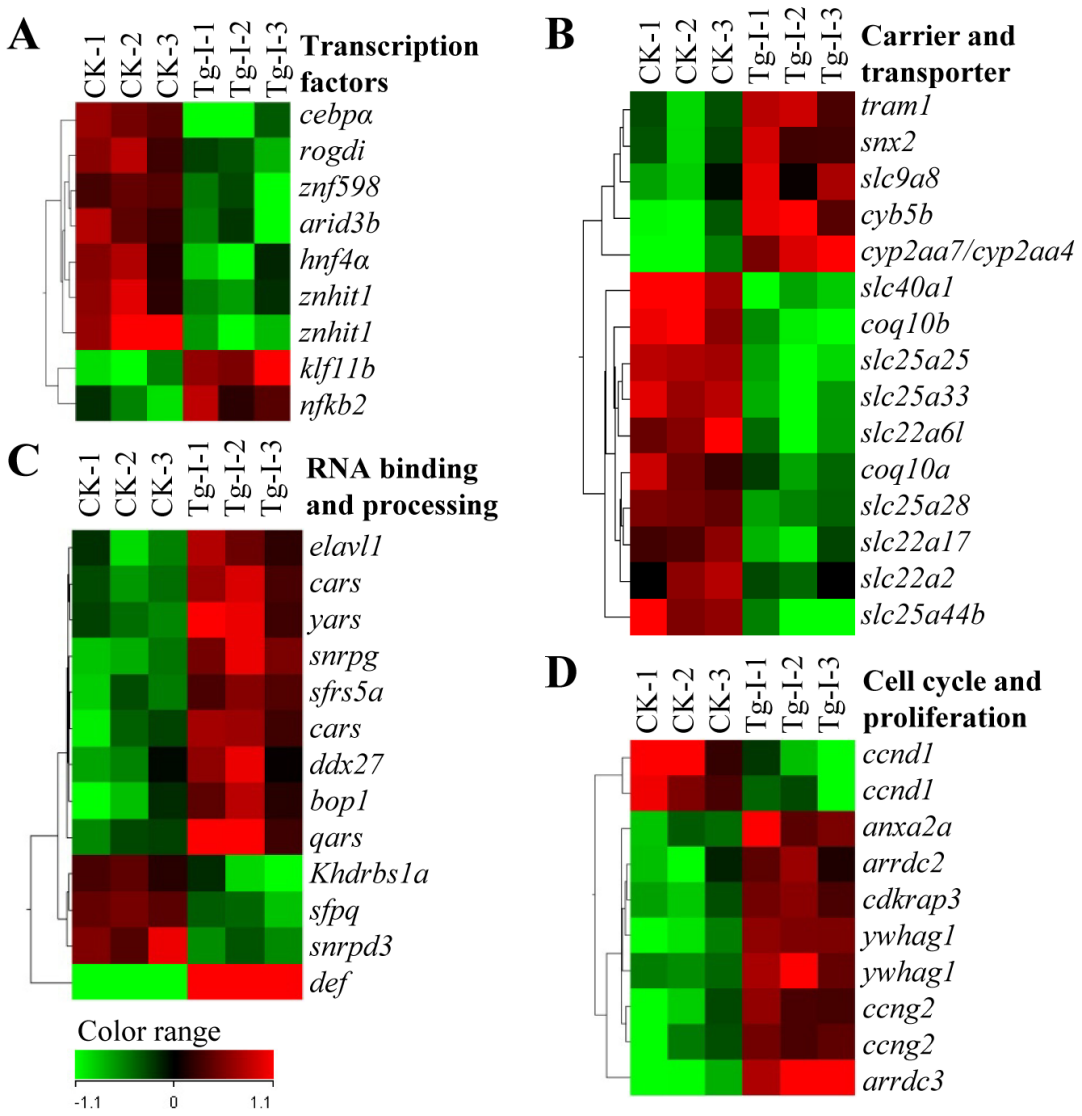
Expression of genes in specific functional pathways was altered in a coordinate way in the liver of *Tg-I*. (**A–D**) Expression of genes in categories for encoding transcription factors (**A**), carrier and transporter (**B**), RNA bidning and processing (**C**), and cell cycle and proliferation (**D**) were found to be coordinately regulated by Def over-expression. CK: wild type adult liver control.

We then summarized genes up- or down-regulated in each of the 11 categories with the purpose to find out the pathway(s) coordinately affected by Def over-expression in the liver in *Tg-I*. Data analysis revealed that only eight transcription factor genes were expressed differently in the liver between *Tg-I* and the WT fish. Interestingly, six out of the eight liver transcription factor genes, namely *cebpα*, *rogdi*, *znf598*, *arid3b*, *hnf4α* and *znhit1* were down-regulated (Figure 6A). *hnf4α* and *cebpα* were decreased by 2.4-fold and 3.6-fold in *Tg-I*, respectively in our microarray analysis, and their expression reduction was further validated by qPCR analysis (Figure 5A).

Hnf4α and C/EBPα are two well-known liver key transcription factors that control the expression of large amount of liver genes [24], [25]. Therefore, down-regulation of *Hnf4α* and *C/EBPα* is expected to cause down- or up-regulation of their target genes which are normally up- or down-regulated by them. Bioinformatic analysis of the 60 down-regulated functional known genes in *Tg-I* revealed that, in addition to the 6 transcription factor genes mentioned in the above, many of the metabolic enzyme genes (15 genes) and solute carrier/transport genes (10 genes) (Figure 6B) were collectively down-regulated (Table S2). On the other hand, among the 77 up-regulated genes, genes in the categories of RNA binding and processing (9 genes) (Figure 6C), cell cycle and proliferation (7 genes) (Figure 6D), energy metabolism (7 genes), chaperones (5 genes), cytoskeleton related (6 genes), non-catabolic enzymes (12 genes) and calcium ion binding (6 genes) were collectively up-regulated in *Tg-I* (Table S2). Considering the fact that liver is a relatively homogenous tissue, this observation strongly suggests that Def regulates specific functional pathways in the liver.

### Def over-expression causes disorganization of intrahepatic structure

**Figure 7:**
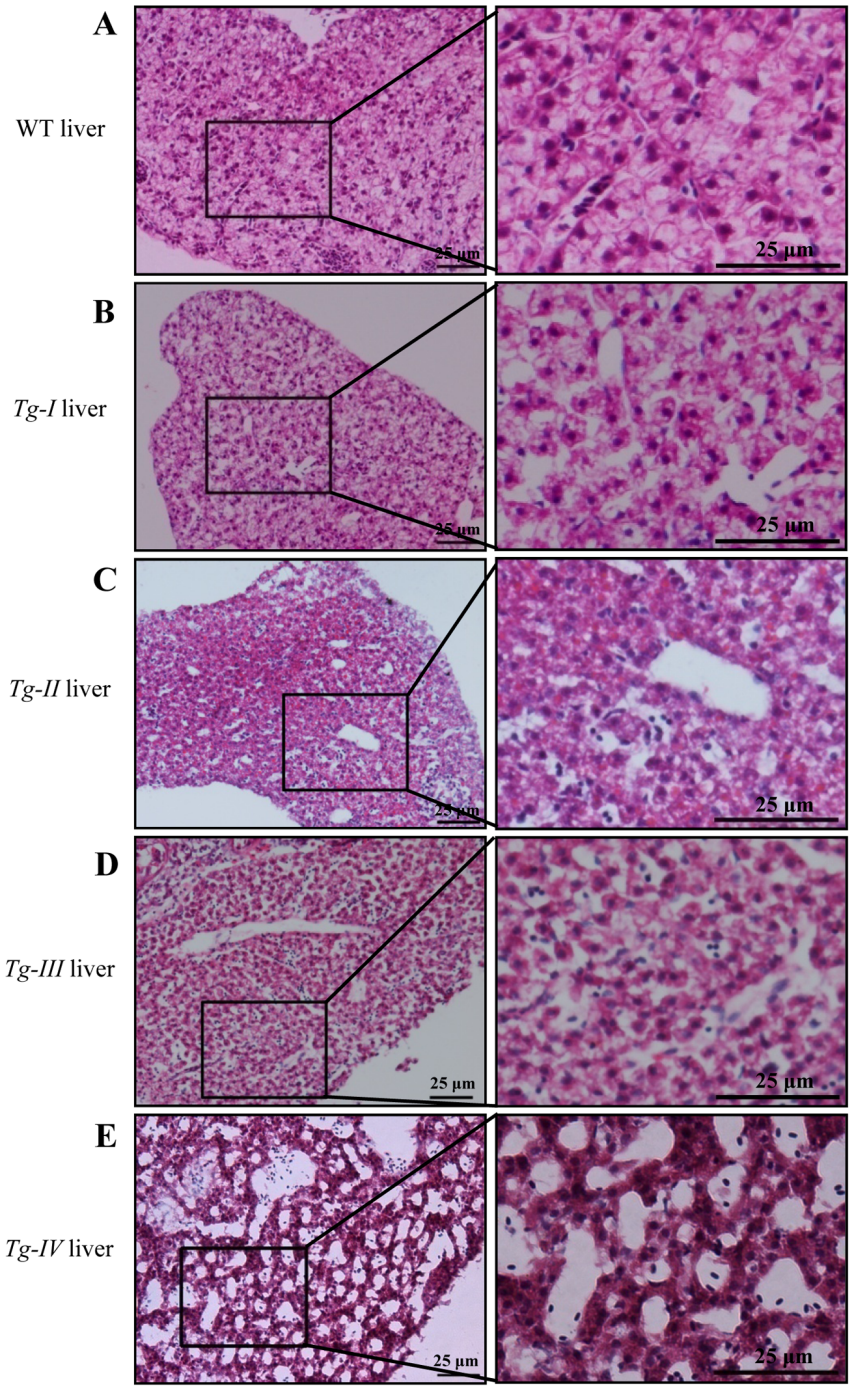
Def over-expression disrupts the intrahepatic structure. (A–E) H&E staining of the liver tissues from 3 months old fish of WT (A), *Tg-I* (B), *Tg-II* (C), *Tg-III* (D) and *Tg-IV* (E) showed that hepatocytes were loosely connected to each other and large gaps was formed in all four lines when compared to the liver tissue in the WT control. Panels on the left: view of the liver tissue at low power of magnification; panels on the right: high power of magnification view of the region boxed in the corresponding panels on the left as indicated.

Up-regulation of genes involved in cell cycle and proliferation (mainly related to cell cycle arrest) (Figure 6D) and genes related to chaperone and cytoskeleton and others suggest that the proliferation and interconnection of hepatocyes might be affected in *Tg-I*. Indeed, analysis of H&E stained liver sections from 3-month-old WT and *Tg-I* fish showed that cells in the *Tg-I* liver loosened in cell-cell junction and failed to form regular patterns as that observed in the WT fish (Figure 7A and B). Furthermore, the liver in *Tg-I* had increased number and size of gap regions within when compared to the liver in a wild type fish (Figure 7B).

**Figure 8:**
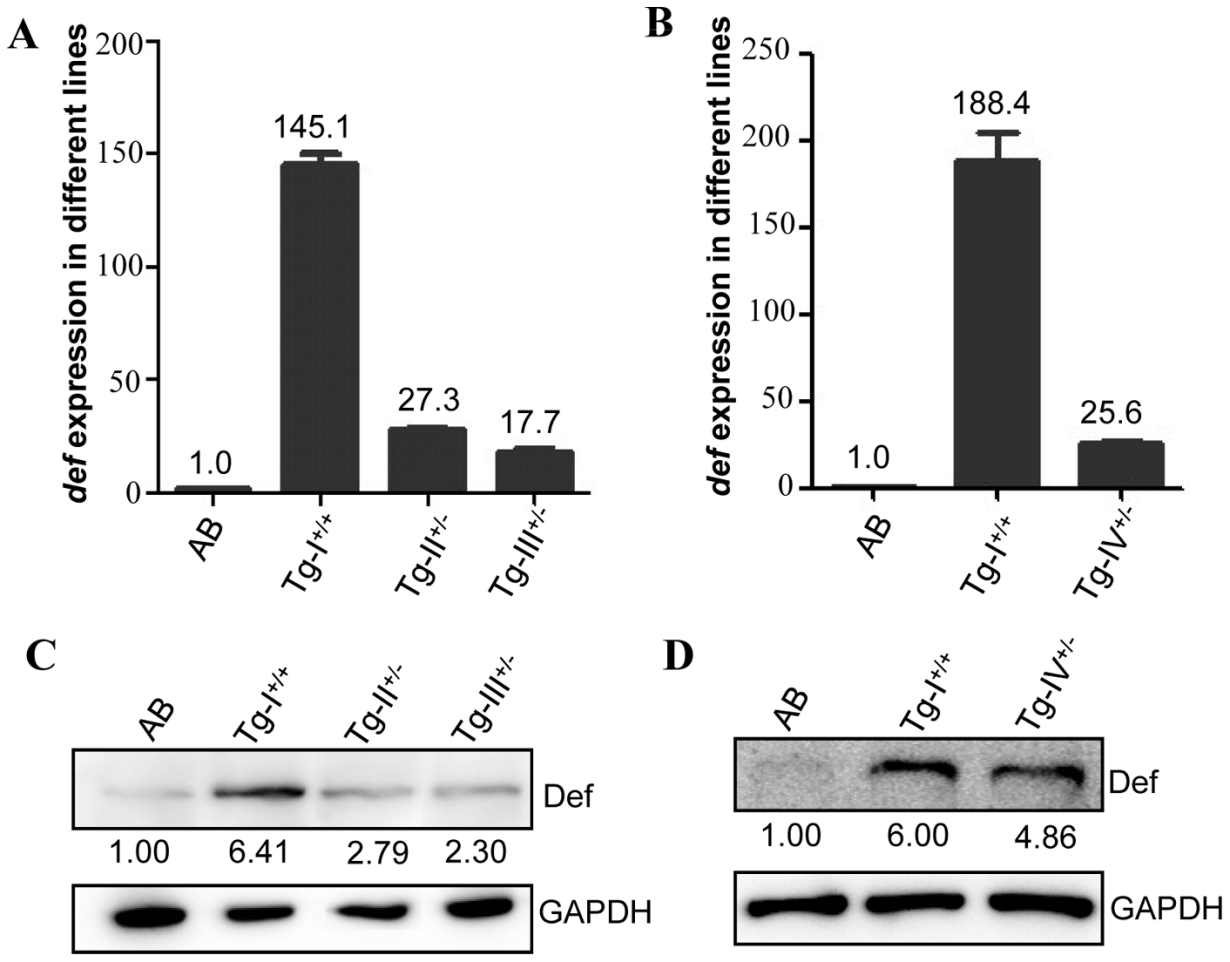
Levels of *def* transcripts and Def protein in the adult liver of *Tg-I*, *Tg-II*, *Tg-III* and *Tg-IV*. (A–D) The *def* transcript levels in *Tg-I*, *Tg-II* and *Tg-III* (A) or in *Tg-I* and *Tg-IV* (B) were determined by qPCR and Def protein levels by western blotting using an anti-Def monoclonal antibody (C and D). Interestingly, while the transcriptional expression of *def* showed a great range of difference in different genetic background (145, 27, 18 and 26 folds higher in *Tg-I*, *Tg-II*, *Tg-III* and *Tg-IV*, respectively, than in the WT) (A and B) the protein levels in these three lines did not differ in a corresponding scale (6.4, 2.8, 2.3 and 4.9 folds higher in *Tg-I*, *Tg-II*, *Tg-III* and *Tg-IV*, respectively, than in the WT) (C and D).

**Figure 9:**
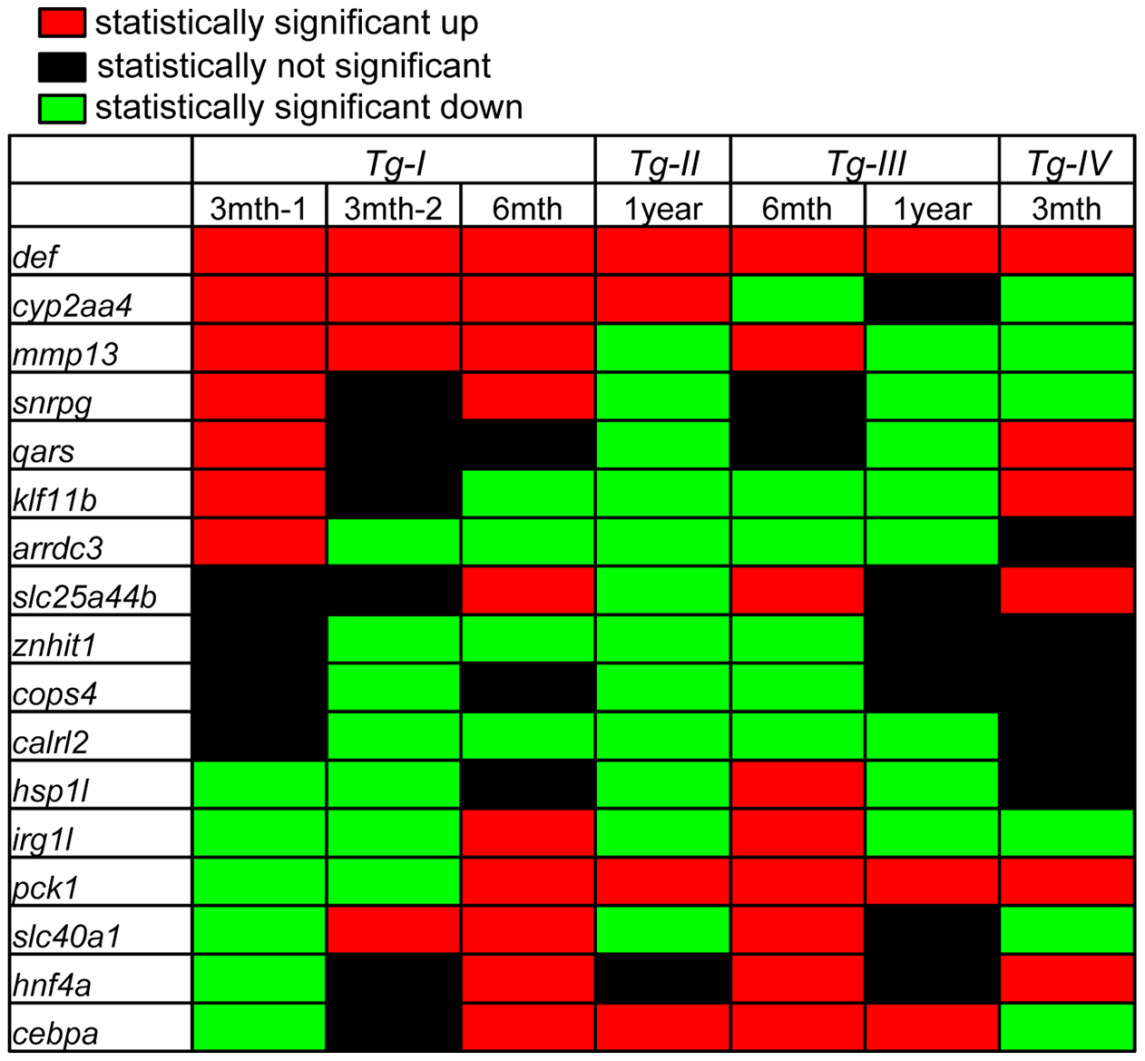
Summary of the expression changes of the 16 genes in the adult livers of four transgenic lines with respect to that in the WT liver. The summary was based on the qPCR data shown in Figure 7. Notably, the data showed that the expression of these 16 genes in *Tg-II^+/−^*, *Tg-III^+/−^* and *Tg-IV^+/−^* did not follow the trend or even opposite changes when compared with that observed in *Tg-I*. Surprisingly, the expression of these 16 genes in different batches of *Tg-I* at the same age were also different.

In order to exclude the possibility that this abnormal adult liver phenotype in *Tg-I* could be due to positional effect (e.g disrupt a gene or affect the expression of adjacent genes by the insertion), we analyzed H&E stained liver sections from the remaining three independent transgenic lines *Tg-II^+/−^*, *Tg-III^+/−^* and *Tg-IV^+/−^*. We first performed the qPCR analysis and found that *def* expression was approximately 145, 27, 18 and 26 folds higher in Tg-I^+/+^, *Tg-II^+/−^*, *Tg-III^+/−^* and *Tg-IV^+/−^*, respectively (Figure 8A and B). Interestingly, western blot analysis showed that the protein levels in these four independent transgenic lines did not differ in a corresponding scale to that of their transcripts and showed 6.4, 2.8, 2.3 and 4.9 folds higher in Tg-I^+/+^, *Tg-II^+/−^*, *Tg-III^+/−^* and *Tg-IV^+/−^*, respectively, than its expression in the WT liver (Figure 8C and D). Surprisngly, qPCR analysis of the same 16 genes listed in Figure 5A showed that the expression of these genes in *Tg-II^+/−^*, *Tg-III^+/−^* and *Tg-IV^+/−^* at different age or the same age did not follow the trend or even opposite changes when compared with that observed in *Tg-I* (Figure 5B–D, Figure 9). We also noticed that even in different batches of *Tg-I* at the same age the expressions of these 16 genes were also different (Figure 9). Despite the discrepancy in gene expression among different Def over-expression transgenic lines, histological analysis showed the adult liver of *Tg-II^+/−^*, *Tg-III^+/−^* and *Tg-IV^+/−^* exhibited similar phenotype (Figure 7C–E) as that observed in *Tg-I* (Figure 7B).

## Discussion

The alimentary tract comprises functional distinct organs each contains specific cell types. From the view of developmental biology, the key question is how specification of different organs and interconnection of these organs to form a complete digestive system is achieved during the process of organogenesis. Three groups of factors are believed to contribute to this process: organ specific factors that define the identity of a specific organ, pan-endodermal factors that coordinate the development of different organs to form a functional digestive system, and signaling molecules from surrounding mesoderm cells that mediate the coordination. Def expression is enriched in the digestive organs, raising a question as to how Def performs its function in different organs. To address this question, it is ideal to express Def in a specific digestive organ in the background of *def^hi429^*. In this way we could examine 1) whether the specific organ development can be restored to normal, and 2) whether it will alter the cell fate in the organs expressing Def. To address this question we generated the transgenic fish *Tg(fabp10a:def)* in which *def* is specifically expressed in the liver. Our result showed that the *def* transgene in *Tg-I* only rescued the liver but not intestine and exocrine pancreas development in *def^hi429^*. Meanwhile, the liver of *Tg-I* expressed the liver marker *fabp10a* but not the intestinal marker *fabp2* and exocrine pancreatic marker *trypsin*. These data demonstrated that Def functions as a cell autonomous factor to regulate the development of digestive organs although it appears not to be a key cell fate determinant.

Previous reports showed that Def homologs in yeast and *Arabiopsis* are nucleolar proteins and are involved in the processing of 18S but not 28S rRNA intermediate precursors [12]–[14]. We wondered whether continuous over-expression of Def in *Tg-I* will affect the processing of 18S rRNA. To our surprise, we found that processing of 18S rRNA precursor in the adult liver of *Tg-I* was affected as that observed in the yeast mutant *Utp25^−/−^* and *Arabidopsis* mutant *nof1*, suggesting that the level of Def must be kept in a narrow range because too little or too much both will affect the processing of rRNA precursors mediated by Def. Loss-of-function of Def leads to hypoplastic digestive organs, it is of our great interest to find out whether over-expression of Def would promote liver development in our future studies.

By comparing global gene expression profiles we previously reported that 141 genes were down-regulated and 23 genes up-regulated in the *def^hi429^* mutant [11]. However, that experiment was performed using total RNA extracted from whole embryos at 5 dpf, therefore the data cannot help us to identify specific pathways in a specific organ based on down-regulated genes because of heterogeneity of origin of the total RNA used. The liver is considered to be a relatively homogenous organ because it contains limited types of cells, with majority (>60%) being hepatocytes. Therefore, over-expressing Def in the liver will not only allow us to study Def's role in the liver development but also serves as a model to define Def's role in regulating gene expression. We compared the global gene expression profiles in the adult liver between *Tg-I* and WT. We found that the *def* transgene in *Tg-I* altered a fraction of genes that can be clearly classified into a few distinct pathways in the liver. Among these pathways, we noticed that nine genes related to rRNA binding and processing (namely *elavl1*, *cars*, *yars*, *snrpg*, *sfrs5a*, *cars*, *ddx27*, *bop1*, *qars*) were up-regulated. This indicates that Def might not only directly regulate rRNA processing as a component of the SSU processome but also affect rRNA homeostasis through other genes and pathways.

It is well-known that the expression of liver-specific and/or -enriched genes are under the control of a network formed by transcription factors including hepatic nuclear factors HNF1, HNF3, HNF4α, HNF6, and C/EBPα (CCAAT/enhancer binding protein) etc [26]–[32]. Therefore, down-regulation of *hnf4α* and *C/EBPα* provides a logic explanation why majority of genes in the categories of catabolic enzyme and solute carrier/transporter were down-regulated in the liver of *Tg-I*.

Analysis of the adult liver revealed that the LBR is reduced significantly in *Tg-I*. Histology study showed that hepatocytes appeared to be loosely connected and there were apparent distended gap regions inside the liver mass in all four independent transgenic lines as well. These results suggest that over-expression of Def in these four transgenic lines not only affects the processing of 18S rRNA precursors but also changes the structural integrity of the adult liver. The structural abnormality of the adult liver in *Tg(fabp10a:def)* might be related to the up-regulation of genes involved in the pathways of cell cycle and proliferation, cytoskeleton related, chaperones and calcium ion binding, and to down-regulation of genes in the pathways of cell adhesion and migration, solute carrier/transporter and DNA remodeling factors. However, one has to be cautious in linking the gene expression profiles with the abnormal liver structure in this specific case since we found that the up- or down-regulated genes in one transgenic line was not the case or even opposite in another transgenic line. This discrepancy might be due to the timing of RNA sampling since the liver structure in all four transgenic lines has already accumulated pathologically identifiable disruptions at 3 months which, conceivably, would result in highly variable gene expression profiles in different transgenic lines at this time point due to different degrees of damages to the hepatocytes. We hypothesize that Def over-expression initially causes changes in expression of certain key genes that are essential to make a perfect liver in all transgenic lines. Changes in the expression of these key genes will ultimately disrupt the liver structure within a time period which, depending on the level of Def expressed, is different in duration for different transgenic lines. Apparently, a key task in the future is to determine the time point when the liver structure just displays abnormality for each independent transgenic lines and then to use this time point or prior to this time point for RNA sampling.

In conclusion, the generation of the *Tg(fabp10a:def)* transgenic fish has allowed us to study Def's function both in the embryonic and adult liver. We found that while Def is essential for the organogenesis of the liver at the embryonic stage its continuous high expression in the adult liver is harmful, leading to structural abnormality of the liver. Therefore, Def protein level must be tuned to an appropriate level to maintain the normal function of the liver. However, we currently do not know whether alteration of gene expression profiles in the *Tg-I* liver is due to aberrant processing of 18S rRNA precursors or to Def's other function. Neither we know whether Def works by its own or team up with other factors to perform its function. Future efforts on addressing these questions will no doubt help us to understand more about the role of the nucleolus in organogenesis of digestive organs.

## Supporting Information

Table S1 **List of primers used for qPCR.**
(XLS)

Table S2 **Classification of genes up- and down-regulated in** ***Tg(fabp10a:def)-I*** **.**
(XLS)
